# Supplementary material for: Use of lay vaccinators in animal vaccination programmes: A scoping review
Source: PLoS Negl Trop Dis. 2021 Aug 10;15(8):e0009691. doi: 10.1371/journal.pntd.0009691 (PMC8378730; doi:10.1371/journal.pntd.0009691)
Supplement: Attachment — Submitted filename: MS Review PLOS.docx [file pntd.0009691.s001.docx]

Review of Manuscript Number PNTD-D-21-00376

Use of lay vaccinators in animal vaccination programmes: A scoping review.

The reviewer agrees with the authors that there is a growing interest in and need for the use of CAHWs in veterinary service delivery and that this can be facilitated by improving the enabling environment for their integration into the veterinary workforce, notably with particular attention paid to the legal and regulatory framework and improved, harmonized training. In this regard, this scoping review is a welcome addition to the literature on CAHWs. The reviewer supports acceptance of the paper following some minor revisions based on the comments below.

183. Perhaps use the term facilitating factors instead of facilitators throughout the manuscript, as ‘facilitator’ may be confusing, as facilitators are often thought of as individual persons rather than supporting processes. Indeed, in some places the term facilitating factors is used, but not consistently

214. in the box on the right, the term grey literature is used to identify the source of three evaluated papers. This requires some explanation in the methods section, as currently there is no mention of the grey literature. Where did these three papers come from? Why were there not more considered from the grey literature?

247. In the table, the abbreviation NR is used but is not included in the list of abbreviations starting on line 247. Please define and include it.

269. Use the term “Lay Animal Vaccinators (LAV)” in the title of the table for convenience and clarity rather than including the acronym at the bottom of the table. Same for table on line 316.

269. Later in Table 3 in the section on Remuneration Arrangements, there is a typo. Batter should be barter.

329. The term ‘no significant differences’ is used. This is usually reserved for cases of actual statistical analysis where no statistically significant difference is identified. It does not appear that any statistical analysis was done in this study.

330. In the first row of Table 5, second column, the word ‘assurance’ is used but the meaning is not clear to the reviewer in the context uses. Please replace or clarify.

340. It would be helpful to have some further explanation or clarification of who other community-level actors might be to better understand how their training contributed to project success. This could be done here or in the discussion section.

352. Similar to the comment above, some additional explanation of what is meant by ‘competing financial interests of vaccinators and community leaders” as it is not clear to the reviewer what the financial interests of the community leaders might be in this regard.

374-377. The reviewer feels that rather than an appendix including all the projects, a Table 7 be added structured similar to the preceding tables that lists the implemented solutions and identifies the papers by citation that include those solutions.

389-390. The authors state that “review showed that lay animal vaccinators generally performed well in delivering animal health services to their communities. Given the evidence available, the reviewer believes this statement is too broad and should focus specifically on vaccinations by saying for example, ‘delivering animal vaccinations’ or delivering preventive animal health services. The reason is that some projects allowed LAVs to administer antibiotics and other medications and no evidence is provided that these interventions were done properly or had positive outcomes, nor was it indicated that any of the projects that included information about curriculum and training included anything on clinical pharmacology or the proper use of antibiotics.

415. The reviewer is not aware of the availability of thermostable foot and mouth disease vaccines. If this is a new development, perhaps a reference citation should be included.

492. Similar to the comment for lines 389-390, the reviewer feels that it is not appropriate here to say that “lay animal vaccinator schemes have been appreciably deployed to treat a wide range of animal diseases. It would be more accurate to say, ‘prevent’ rather than ‘treat’ as vaccines have a prophylactic function, not a therapeutic one.

516 0nward. The citations need serious attention. There does not seem to be a consistent format to their presentation, and there are numerous typos and omissions of citation elements throughout. The entire list needs to be carefully reviewed, corrected and standardized.
