# Supplementary material for: Use of lay vaccinators in animal vaccination programmes: A scoping review
Source: PLoS Negl Trop Dis. 2021 Aug 10;15(8):e0009691. doi: 10.1371/journal.pntd.0009691 (PMC8378730; doi:10.1371/journal.pntd.0009691)
Supplement: Attachment — Submitted filename: Letter to the Editors_Response to reviewers comments.docx [file pntd.0009691.s002.docx]

Dear Editors,

**Response to yours and reviewers’ comments**

Use of lay vaccinators in animal vaccination programmes: A scoping review (PNTD-D-21-00376).

Thank you very much for the opportunity to respond to reviewers’ comments in a revised submission of our manuscript. We thank the reviewers for constructive comments which we have now addressed, and we consider have resulted in an improved manuscript. We respond to your own comments first and then to each referee in turn. Page numbers refer to those in the revised manuscript with tracked changes.

**Editors’ comments**

| Comment | Response |
| --- | --- |
| 183. Perhaps use the term facilitating factors instead of facilitators throughout the manuscript, as ‘facilitator’ may be confusing, as facilitators are often thought of as individual persons rather than supporting processes. Indeed, in some places the term facilitating factors is used, but not consistently | Thank you for your suggestion as to how to use the term ‘facilitator’. We found 8 instances where the word ‘facilitator’ was used and have replaced it with the phrase ‘facilitating factors’ (lines 186-7, 191, table 1, 337, 342, 451-2). |
| 214. in the box on the right, the term grey literature is used to identify the source of three evaluated papers. This requires some explanation in the methods section, as currently there is no mention of the grey literature. Where did these three papers come from? Why were there not more considered from the grey literature? | Thank you for your suggestion. We recognize that that is not the most appropriate phrase to designate the sources of those papers and it has now been removed. These papers were identified from reference lists and we have now corrected this, removing ‘grey literature’ from the box in the figure and stating their sources in the methods section (line 168-9). |
| 247. In the table, the abbreviation NR is used but is not included in the list of abbreviations starting on line 247. Please define and include it. | The abbreviation ‘NR: Not Reported’ is now defined and included in the list of abbreviations (line 252). |
| 269. Use the term “Lay Animal Vaccinators (LAV)” in the title of the table for convenience and clarity rather than including the acronym at the bottom of the table. Same for table on line 316. | We have now used the term and abbreviation in the titles, and will serve as a reference to the abbreviation in the rest of the tables. The definition of the abbreviation is removed from the end of the tables (lines 272-3, 321-2). |
| 269. Later in Table 3 in the section on Remuneration Arrangements, there is a typo. Batter should be barter. | We have corrected this typo. |
| 329. The term ‘no significant differences’ is used. This is usually reserved for cases of actual statistical analysis where no statistically significant difference is identified. It does not appear that any statistical analysis was done in this study. | We have reworded the statement to show we simply are reporting the findings reported from those papers we found and not from our own analysis (lines 332-6). |
| 330. In the first row of Table 5, second column, the word ‘assurance’ is used but the meaning is not clear to the reviewer in the context uses. Please replace or clarify. | We have qualified the phrase to indicate ‘farmer assurance’, which means farmers became more confident in investing in animal assets (Table 5). |
| 340. It would be helpful to have some further explanation or clarification of who other community-level actors might be to better understand how their training contributed to project success. This could be done here or in the discussion section. | We have added further specific information for clarification (line 348). |
| 352. Similar to the comment above, some additional explanation of what is meant by ‘competing financial interests of vaccinators and community leaders” as it is not clear to the reviewer what the financial interests of the community leaders might be in this regard. | We have added examples to clarify ‘competing financial interests’ (lines 362-8). |
| 374-377. The reviewer feels that rather than an appendix including all the projects, a Table 7 be added structured similar to the preceding tables that lists the implemented solutions and identifies the papers by citation that include those solutions. | Thank you for this suggestion. A table 7 is now added and presents information on proposed or implemented solutions to the challenges faced by lay animal vaccinator programmes (lines 392-394, Table 7). |
| 389-390. The authors state that “review showed that lay animal vaccinators generally performed well in delivering animal health services to their communities. Given the evidence available, the reviewer believes this statement is too broad and should focus specifically on vaccinations by saying for example, ‘delivering animal vaccinations’ or delivering preventive animal health services. The reason is that some projects allowed LAVs to administer antibiotics and other medications and no evidence is provided that these interventions were done properly or had positive outcomes, nor was it indicated that any of the projects that included information about curriculum and training included anything on clinical pharmacology or the proper use of antibiotics. | We have narrowed the scope of the statement to ‘delivering animal vaccinations’ (line 409). |
| 415. The reviewer is not aware of the availability of thermostable foot and mouth disease vaccines. If this is a new development, perhaps a reference citation should be included. | We initially included foot and mouth disease vaccines in the discussion, as research methods for generating thermostable vaccines have been described for foot and mouth disease. However, we also cannot find any reference to a thermostable vaccine that is currently available and have removed this from the text (line 434). |
| 492. Similar to the comment for lines 389-390, the reviewer feels that it is not appropriate here to say that “lay animal vaccinator schemes have been appreciably deployed to treat a wide range of animal diseases. It would be more accurate to say, ‘prevent’ rather than ‘treat’ as vaccines have a prophylactic function, not a therapeutic one. | We have replaced the word ‘treat’ with ‘prevent’ to more accurately reflect the expected outcomes of their work (line 525). |
| 516 0nward. The citations need serious attention. There does not seem to be a consistent format to their presentation, and there are numerous typos and omissions of citation elements throughout. The entire list needs to be carefully reviewed, corrected and standardized. | We have corrected and standardized the references, ensuring that each reference is formatted in line with PLOS reference formatting guidelines (lines 550-769). |

Reviewer #1

| Comment | Response |
| --- | --- |
| I worry that the search criteria used to identify studies was too narrow (Line 161). I recognise that highlighting individual publications as examples in such an exercise is unhelpful where they may have been omitted in the review methods described, but from the rabies literature alone the following recent paper would appear to not have met the initial search criteria stated in Line 161 due to not having used the terms “vaccinator” or “animal health worker”, despite appearing to align with the scope of the review: “Volunteer based approach to dog vaccination campaigns to eliminate human rabies: Lessons from Laikipia County, Kenya” (https://doi.org/10.1371/journal.pntd.0008260). This paper may have fallen out in the subsequent screening, but my concern is that if it were not captured in the initial search criteria then many relevant publications may have been missed. Apologies if I have misinterpreted the current search methodology and papers such as this were  captured and excluded. | Thank you for drawing our attention to this, but we believe the original search did address the main point made by the reviewer i.e. the search terms did include reference to ‘lay OR community-based OR volunteer AND "animal health worker" OR vaccinator*’. The term ‘vaccinator’ was used with the Boolean operator ‘*’ to capture other forms of the word (vaccination and vaccinating) (lines 163-4).  Our search did pick up the paper by Ferguson et al., 2020 and this is definitely an interesting paper. However, it did not meet our inclusion criteria as the volunteers were animal health or wildlife professionals, and our objective was to identify programmes which involved lay (non-professional) persons in animal vaccination. We do think there are important insights from the Ferguson et al. 2020 paper, and have referenced the paper now in the discussion (lines 497-8). |
| The discussion is well structured and largely well balanced, highlighting the benefits and challenges around the use of lay vaccinators. Clearly there is a huge need to to expand the available workforce for vaccine delivery across LMICs to improve human and animal health, however there is one aspect which I feel needs further consideration in the discussion.  Concerns around lay vaccinators potentially doing harm is mentioned in the discussion (Line 449), in the misuse of drugs, however the possibility of over-reach in their clinical duties is not discussed. This is alluded to as a finding from the literature in Line 313, where it states they take part in dog population control activities and sterilisation, presumably meaning lay persons performing surgical procedures. It would be important to flag the considerable animal welfare issues resulting from untrained and poorly equipped persons undertaking certain veterinary duties and the challenges in monitoring such practices in the settings in question. The need for regulation is well discussed, but the perspective of devolving veterinary responsibilities without enforceable regulation is not highlighted, along with the potential for this to undermine the sometimes frail existing veterinary frameworks established to date. Largely these concerns are reflected in the authors' emphasis on the need for involvement of the veterinary sector, training, regulation and strong OIE guidelines etc, however inclusion of the risks of devolution without successful regulation would be an important addition. Examples are given of where lay vaccinators have been used in the developed world to rapidly expand the vaccinator workforce, however well-established regulatory frameworks have enabled this to be done in a controlled manner. Applying the same principles in rural areas of LMICs carries vastly different challenges in communicating and enforcing such regulations and I feel that this should be given greater weight in the discussion and conclusion. | Thank you for these constructive comments. We entirely agree that our use of the phrase ‘dog population control’ in the results section could imply that lay vaccinators performed activities as sterilizations or surgical procedures. We have now clarified the text to indicate that the role the lay persons played was to encourage responsible dog ownership and management to ensure dog not interact freely and breed uncontrollably (lines 317-8).  On the issue of broadening the discussion to other roles played by lay animal health workers, we wish to indicate that this paper was focused specifically on animal vaccination, and we did not review literature on other types of interventions. We therefore consider it beyond the scope of this paper to generalize much beyond animal vaccination (with dog population management included as this is so often linked with rabies vaccination). We are also hesitant to broaden the discussion as this may have the potential to be mis-interpreted as advocacy for greater involvement of lay person in interventions that require specialized skills and training.  The statement on the potential to cause harm is further strengthened in lines 441-5.  We have, however, mentioned in the discussion and conclusion why devolving veterinary roles to lay persons can be difficult to regulate in LMICs, and much attention should be given to strengthening the regulatory systems where they are implemented (lines 457-462, 533-4). |

Reviewer #2

| Comment | Response |
| --- | --- |
| My main concern with the paper (albeit a small concern) is that the sampling method can be expected to have a strong bias towards programs that were successful. The authors admit this selectively in lines 394-396 and 485. I therefore find it hard to trust the quantitative conclusions drawn (not only the statement preceding line 394).  I propose including a general statement on the limitations of the sampling method and consequently all the reported proportions. | Thank you for the observation. We agree that there are likely to be publication biases in relation to reporting studies with positive outcomes. We have now strengthened the statements about the limitations of the sampling method (line 510-13)  We have added a statement on limitations of the study in relation to validity and generalizability of findings (lines 483-91). |
| Some of the headings of Figure 1 have been cropped (Identificatio_, Eligibili__). | We have replaced the boxes and the headings. |
| Reviewer #2: In my view the paper has something to offer to the scientific community, namely that lay people present an underutilized resource.  I think that the paper could benefit from the inclusion of some evidence from outside of the scientific literature (consider personal communications or press reports). This could strengthen some of the stated arguments. | We are pleased that the reviewer considers that lay people present an underutilized resource. However, we sense that because of widespread resistance to these approaches, professional veterinarians and people outside the profession have been reluctant to publicly express this opinion. We therefore do not have statements to strengthen some of the stated arguments. A major objective of carrying out this study was to present a formal and robust evaluation of the literature and hope that our results will give people confidence to discuss these approaches more openly. |
